# Prion Pathogenesis Is Faithfully Reproduced in Cerebellar Organotypic Slice Cultures

**DOI:** 10.1371/journal.ppat.1002985

**Published:** 2012-11-01

**Authors:** Jeppe Falsig, Tiziana Sonati, Uli S. Herrmann, Dino Saban, Bei Li, Karina Arroyo, Boris Ballmer, Pawel P. Liberski, Adriano Aguzzi

**Affiliations:** 1 Institute of Neuropathology, Zürich, Switzerland; 2 Laboratory of Electron Microscopy and Neuropathology, Department of Molecular Pathology and Neuropathology, Medical University of Lodz, Lodz, Poland; University of Edinburgh, United Kingdom

## Abstract

Prions cause neurodegeneration in vivo, yet prion-infected cultured cells do not show cytotoxicity. This has hampered mechanistic studies of prion-induced neurodegeneration. Here we report that prion-infected cultured organotypic cerebellar slices (COCS) experienced progressive spongiform neurodegeneration closely reproducing prion disease, with three different prion strains giving rise to three distinct patterns of prion protein deposition. Neurodegeneration did not occur when PrP was genetically removed from neurons, and a comprehensive pharmacological screen indicated that neurodegeneration was abrogated by compounds known to antagonize prion replication. Prion infection of COCS and mice led to enhanced fodrin cleavage, suggesting the involvement of calpains or caspases in pathogenesis. Accordingly, neurotoxicity and fodrin cleavage were prevented by calpain inhibitors but not by caspase inhibitors, whereas prion replication proceeded unimpeded. Hence calpain inhibition can uncouple prion replication from its neurotoxic sequelae. These data validate COCS as a powerful model system that faithfully reproduces most morphological hallmarks of prion infections. The exquisite accessibility of COCS to pharmacological manipulations was instrumental in recognizing the role of calpains in neurotoxicity, and significantly extends the collection of tools necessary for rigorously dissecting prion pathogenesis.

## Author Summary

Transmissible spongiform encephalopathies (TSEs) are a group of fatal protein misfolding diseases causing neurodegeneration *in vivo*. TSEs are unique in that the infectious agent termed ‘prion’ consists of a misfolded protein lacking sequence specific nucleic acids. Prion-infected cultured cells do not develop visible pathological changes, and this has hampered mechanistic studies of prion-induced neurodegeneration. Here, we have developed a prion-induced neurodegeneration model that uses cultured slices of living brain tissue. Such slices display all the classical hallmark of prion disease, namely prion replication, inflammation, spongiform changes and neurodegeneration. Neurotoxicity is blocked by anti-prion drugs by reducing prion replication. We demonstrate for the first time an involvement of calcium-regulated cysteine proteases called calpains in driving neurotoxicity. We find that the proteolytic processing of the calpain substrate is induced by prion infection and blocked by calpain inhibitors without prion replication being affected. The assay system developed here allows for precise dissection of the mechanisms of prion-induced degeneration with pharmacological means.

## Introduction

Transmissible spongiform encephalopathies (TSE) are inexorably fatal neurodegenerative disorders caused by prions [1] which consist of PrP^Sc^, a protease-resistant isoform of the normal cellular prion protein PrP^C^. Accordingly, *Prnp* ^o/o^ mice lack PrP^C^, cannot generate PrP^Sc^, and withstand prion inoculation [2]. PrP^Sc^ forms aggregates that grow by recruiting PrP^C^ and whose breakage underlies prion replication [3]. The hallmarks of TSEs include PrP^Sc^ deposition and progressive brain damage. *Prnp* ^o/o^ mice show mild phenotypes and no TSE [4]–[7], indicating that TSEs are not caused by loss of PrP^C^ function. Several observations suggest that extracellular deposition of PrP^Sc^ is intrinsically innocuous [8]–[10], whereas neurotoxicity is driven by unknown secondary triggers. A mechanistic dissection of prion neurotoxicity necessitates faithful, experimentally versatile *in vitro* models – yet such models have proven difficult to generate [11], [12].

COCS can be infected with various prion strains [13], with prion titers peaking within 4 weeks. We reported that COCS retain their normal cerebellar architecture and do not experience prion-induced damage within a 1-month observational period. We have now maintained intact organotypic morphology for several months. Under these conditions we observed progressive neurodegeneration starting 5 weeks post-inoculation in prion-infected COCS.

## Materials and Methods

### Ethics statement

All mouse experiments for generation of prion isolates conformed to Swiss law, were performed according to Swiss federal guidelines (‘Ethical Principles and Guidelines for Experiments on Animals’ 3^rd^ edition, 2005) and were approved by the Animal Experimentation Committee of the Canton of Zurich (permit 200/2007). The specific experiments reported in this study were mostly performed in primary cultures and cell lines and for the most part substituted *in vivo* experiments with *ex vivo* experiments.

### Chemicals and mice

All compounds were purchased from Sigma-Aldrich unless otherwise stated. GABA_A_-α6-cre mice were generated on a C57BL/6xCBA background and intercrossed with *Prnp* ^o/o^ mice [5], [14]. Tg37 mice allowing for conditional PrP deletion was generated on a *Prnp* ^o/o^ FVB background [15]. GABA-Aα6-CRE-;loxPrP-tg37 littermates were used as negative controls (PrP^CGC+^). *Prnp* ^o/o^ and *Prnp* ^o/o^;*tg*a*20* ^+/+^ (*tg*a*20*), were on a mixed 129Sv/BL6 background [5], [16]. Rocky Mountain Laboratory strain (RML; passage #6) and 22L prions were amplified in CD1 mice, and 139A prions were amplified in C57BL/6 mice, by intracerebral inoculation into the lateral forebrain of 30 µl 1% (wt/vol) brain homogenate.

### Organotypic brain culture preparation and prion inoculation

Organotypic cerebellar slice cultures, 350 µm thick, were prepared from 10–11 day-old pups according to a previously published protocol [17]. Cultures were inoculated with 100 µg brain homogenate per 10 slices. Brain homogenate was diluted in 2 ml physiological Gey's balanced salt solution (GBSS) (NaCl 8 g l^−1^, KCl 0.37 g l^−1^, Na_2_HPO_4_ 0.12 g l^−1^, CaCl_2_ 2H_2_O 0.22 g l^−1^, KH_2_PO_4_ 0.09 g l^−1^, MgSO_4_ 7H_2_O 0.07 g l^−1^, MgCl_2_ 6H_2_O 0.210 g l^−1^, NaHCO_3_ 0.227 g l^−1^) supplemented with the glutamate receptor antagonist kynurenic acid (1 mM) (GBSSK). Slices were incubated with infectious brain homogenates as free-floating sections for 1 h at 4°C. Slices were then washed twice in 6 ml GBSSK, and 5–10 slices were placed on a 6-well Millicell-CM Biopore PTFE membrane insert (Millipore). After removing any residual buffer, inserts were transferred to a cell culture plate and cultured in “slice-culture medium” (50% vol/vol MEM, 25% vol/vol basal medium Eagle and 25% vol/vol horse serum supplemented with 0.65% glucose (wt/vol), penicillin/streptomycin and glutamax (Invitrogen)). Cultures were kept in a standard cell incubator (37°C, 5% CO_2_, 95% humidity) and the culture medium was exchanged three times weekly. Slices were harvested for protein analyses or fixed for immunocytochemical analysis at various time points.

### Pharmacological treatment of COCS

Drug treatment was initiated 21 days post-inoculation (dpi) in all cases. Drugs were re-added at every medium change. Appropriate drug concentrations were determined by literature search, and we assumed that slice culture uptake of compounds were similar to other cell culture systems. The toxicity of each compound was assessed by measuring NeuN immunoreactivity in parallel on infected and non-infected slices; if toxicity occurred, drugs were retested at a lower concentration. Drug and concentration used were (2S,3S)-*trans*-epoxysuccinyl-L-leucylamido-3-methylbutane ethyl ester (E64d, 15 µM, Bachem), z-Val-Phe-CHO (MDL-28170, 50 µM), N-benzyloxycarbonyl-L-leucylnorleucinal (calpeptin, 100 µM), benzyloxycarbonyl-Asp-Glu-Val-Asp (OMe) fluoromethylketone (zDEVD-fmk, 20 µM), N-acetyl-L-α-aspartyl-L-α-glutamyl-N-(2-carboxyl-1-formylethyl)-L-valinamide (Ac-DEVD-CHO, 20 µM, Promega), benzyloxycarbonyl-Val-Ala-Asp (OMe) fluoromethylketone (zVAD-fmk, 40 µM, R & D Systems). Drug and concentration used for antiprion compounds were: pentosan polysulphate (PPS, 300 ng ml^−1^, kindly provided by Bene pharmachem), quinacrine (1 µM), Congo red (1 µg ml^−1^), amphotericin B (4.5 µg ml^−1^), suramin (50 µM), curcumin (1 µM), cannabidiol (5 µM, Tocris), imatinib mesylate (10 µM, LC Laboratories) and 2,6-dichlorobenzylideneaminoguanidine acetate (guanabenz, 5 µM).

### Misfolded Protein Assay (MPA)

Aggregated PrP was assessed using the Misfolded Protein Assay [18], [19]. Brain homogenate containing 5 µg protein was diluted in 1 ml TBS-T and subjected to affinity-based PrP^Sc^ enrichment using magnetic beads coupled to the peptoid ASR-1 (KKKFKF). Samples were incubated for 1 h at 37°C under permanent agitation (750 rpm), washed, and digested with trypsin (50 µg ml^−1^ in 0.01 M CaCl_2_) for 30 min at 37°C (750 rpm) [20]. Captured PrP was released and disaggregated by adding 75 µl of 0.1 N NaOH (10 min; 750 rpm). After neutralization (30 µl 0.3 M Na_2_H_2_PO4, 5 min, 750 rpm), samples were placed on a magnet to remove the beads, and 150 µl supernatant was analyzed by a PrP ELISA by transferring the samples to POM19-coated ELISA plates [21]. After 1 h incubation at 37°C (30 rpm), plates were washed and POM2-AP was added (1 h, 37°C). After 3 washes, Lumiphos plus substrate (ECL, Amersham) was added, incubated for 30 minutes at 37°C, and plates were read using a chemiluminescence reader. All samples were analyzed at dilutions falling within the logarithmic dose-response range of the calibration curves.

### Quantification of PrPC expression by homogeneous-phase Förster's Resonance Energy Transfer (FRET)

A FRET based assay was established for the purpose of quantifying FL-PrP expression in cerebellar slices. Europium (Eu^3+^) donor and allophycocyanin (APC) acceptor fluorophores were coupled to anti-PrP holoantibodies POM1 and POM2 recognizing the globular domain and the octarepeats, respectively. The donor POM1-Eu^3+^ conjugate is excited at wavelength 340 nm and transfers energy to the acceptor conjugate POM2-APC when the distance between acceptor and donor is <10 nm. POM2-APC then emits light at wavelength 665 nm, which can be measured with a suitable time-resolving spectrofluorimeter. To detect PrP level in homogenates, COCS were lysed; the Eu^2+^-POM1 and APC-POM2 antibody pair was added, measured immediately using a FRET reader and normalized to total protein. Cerebellar slices were lysed in buffer containing 50 mM Tris-HCl pH 8, 0.5% Na deoxycholate, and 0.5% Triton X-100.

### Western blot analysis

COCS were washed twice in PBS. Cerebellar tissue was then scraped off the membrane using 10 µl PBS per slice, and homogenized by trituration using a 30 G syringe, followed by 2×30 s pulses in a sonicator bath. Protein concentration was determined using the bicinchoninic acid assay (Pierce). Samples were adjusted to 20 µg protein in 20 µl and digested with 25 µg ml^−1^ proteinase K in digestion buffer (0.5% wt/vol sodium deoxycholate and 0.5% vol/vol Nonidet P-40 in PBS) for 30 min at 37°C. This protocol allowed specific detection of PrP^Sc^ as shown previously [13]. PK digestion was stopped by adding loading buffer (NuPAGE, Invitrogen) and boiling samples at 95°C for 5 min. Proteins were separated on a 12% Bis-Tris polyacrylamide gel or for higher molecular weight proteins on a 4–12% gradient gel (NuPAGE, Invitrogen) and blotted onto a nitrocellulose membrane. Membranes were blocked with 5% wt/vol Topblock (Fluka) in Tris-buffered saline supplemented with Tween (150 mM NaCl, 10 mM Tris HCl, 0.05% Tween 20 (vol/vol)) and incubated with primary antibodies in 1% Topblock. Primary mouse monoclonal antibodies used were: POM1 mouse IgG_1_ antibody raised against PrP^C^ (anti-PrP^C^) (200 ng ml^−1^), anti-α-fodrin (AA6, 100 ng ml^−1^, Millipore), anti-GAPDH (200 ng ml^−1^, Millipore), and anti-actin (200 ng ml^−1^, Chemicon). Secondary antibody used was horseradish peroxidase (HRP)-conjugated rabbit anti–mouse IgG_1_ (1∶10,000, Zymed). Blots were developed using SuperSignal West Pico chemiluminescent substrate (Pierce) and visualized using the VersaDoc system (model 3000, Bio-Rad). COCS samples for fodrin blots were harvested in PBS with 0.5% DOC, 0.5% NP-40 supplemented with 1 mM AEBSF and complete mini protease inhibitor cocktail (Roche). Because of inefficiencies intrinsic to the tissue slice homogenization procedure, some variation in loading occurred and as a consequence all samples were normalized to GAPDH as a loading control. *In vivo* samples for fodrin blots were homogenized in PBS with 0.32 M sucrose supplemented with 1 mM AEBSF and ‘Complete mini’ protease-inhibitor mix. All samples were normalized to alpha-tubulin. PNGase treatment was performed using a commercially available kit, according to the manufacturer's protocol (New England Biolabs). In brief, 10 µg protein was treated with 2 µl denaturation buffer in a 20 µl reaction and incubated for 15 min at 95°C. A reaction mixture of 2.6 µl G7, 2.6 µl NP-40 (10%), as well as 0.5 µl PNGase was added and samples were incubated for 4 h at 37°C. Samples were then mixed with loading dye, cooked and analyzed by western blotting.

### Histoblots and immunocytochemistry

Histoblot analysis was performed according to a standard protocol using 50–100 µg ml^−1^ PK (30 min, 37°C) [22]. Fresh tissue sections were incubated on PVDF membranes soaked in lysis buffer. After protein transfer, membranes were digested with PK for 4 hours and PrP^Sc^ was detected with antibody POM1 to PrP. The fluorescence Apoptag TUNEL assay was performed on formalin fixed tissue sections according to the manufacturers protocol (Millipore). For immunocytochemistry, the organotypic slices were washed twice in PBS and fixed in 4% formalin overnight at 4°C. Membrane inserts were washed and incubated for 1 h in blocking buffer (0.05% vol/vol Triton X-100 and 3% vol/vol goat serum dissolved in PBS) and incubated with primary antibodies diluted in blocking buffer at 4°C for 3 d. Primary antibodies and concentrations used were ascites fluid of mouse anti–chicken calbindin IgG_1_ antibody (1∶2,000, Swant), rabbit anti-activated caspase 3 (1∶300, BD Biosciences), rabbit anti-human synaptophysin (1∶300, Zymed), rat anti-MBP IgG_2a_ (1∶700, Serotec) and mouse anti-Neuronal Nuclei (NeuN, 1 µg ml^−1^, Millipore). The primary antibodies were detected using Alexa-conjugated secondary antibodies (3 µg ml^−1^, Molecular Probes) and counterstained with 4,6-diamidino-2-phenylindole (dapi) (1 µg ml^−1^). For NeuN morphometry images were recorded at 4× magnifications on a fluorescence microscope (BX-61, Olympus) equipped with a cooled black/white CCD camera and for caspase-3 stains on a Leica SP5 confocal laser scanning microscope using a 63× oil immersion lens. NeuN images were acquired at identical exposure times, and the area of immunoreactivity was determined by morphometry with image analysis software analySIS vs5.0 using identical grey-scale threshold settings for identifying positive pixels. Histology was performed on formalin-fixed, formic-acid decontaminated COCS. Specifically, COCS were dehydrated and embedded in paraffin with their membrane support. The paraffin blocks were then turned by 90 degrees, re-embedded, and cut for conventional histology.

### Proteolysis assays

For caspase-3 activity determinations, slices were harvested in pools (*n* = 20) in PBS, 0.5% DOC, 0.5% NP-40 with 2% β-mercaptoethanol and homogenized by trituration. Homogenates were analyzed immediately for DEVDase activity using caspase 3 fluorometric detection kit (Assay design) and normalized to protein concentration determined by Bradford assay.

### Quantitative PCR

Organotypic slice cultures were prepared and incubated as previously stated. Cultures were washed once with PBS and total RNA was extracted using TRIzol reagent (Invitrogen Life Technologies) according to the manufacturer's protocol. Before cDNA synthesis, residual genomic DNA was removed using the DNA-free kit (Ambion); cDNA was synthesized from 1 mg total RNA with the QuantiTect reverse transcription kit (Qiagen) using random hexamers according to the manufacturer's protocol. We tested for successful cDNA synthesis (+reverse transcriptase) and contamination of total RNA with genomic DNA (−reverse transcriptase) by PCR with primers specific for β-actin (Actb). Quantitative real-time PCR was performed using the SYBR Green PCR Master Mix (Applied Biosystems) on an ABI PRISM 7700 Sequence detector (PerkinElmer). Regulation was calculated relative to untreated wildtype slices after normalization to the Actb signal. The following primer pairs were used: Actb sense, 5′-GAC GGC CAG GTC ATC ACT AT-3′; antisense, 5′-ACA TCT GCT GGA AGG TGG AC-3′. TNF sense, 5′-CAT CTT CTC AAA ATT CGA GTG ACA A-3′; antisense, 5′-TGG GAG TAG ACA AGG TAC AAC CC-3′. MCP-1 sense, 5′-TTA AAA AAC CTG GAT CGG AAC CAA-3′; antisense, 5′-GCA TTA GCT TCA GAT TTA CGG GT-3′. Rantes sense, 5′-ATG CCG ATT TTC CCA GGA CC-3′; antisense, 5′-TTT GCC TAC CTC TCC CTA CTG-3′.

### Electron microscopy

Slices were washed in Na-phosphate buffer, fixed in freshly prepared 2% PFA+2.5% GA in 0.1 M Na-phosphate buffer 0.1 M pH 7.4, postfixed in osmium tetroxide, embedded in epon and examined with transmission electron microscopes Jeol JEM 1011 and 100CX. Each sample grid was divided into 20 equally sized “grid squares” and the number of objects of interest (vacuoles, dystrophic neurites and tubulovesicular structures) was determined for each “grid square” covered by tissue.

### Viability assay

For propidium iodide (PI) incorporation, slices were incubated for 30 min with PI (5 µg ml^−1^) and images were recorded in living tissue using a fluorescent microscope (Axiovert 200) equipped with a cooled CCD camera using a 5× objective. Images were analyzed by morphometry.

### Scrapie cell assay in endpoint format (SCEPA)

Prion-susceptible neuroblastoma cells (subclone N2aPK1) [23] were exposed to 300-µl brain homogenates using 6–12 replicas per dilution in 96-well plates for 3 d. Cells were subsequently split three times 1∶3 every 2 days, and three times 1∶10 every 3 d. After the cells reached confluence, 25’000 cells from each well were filtered onto the membrane of ELISPOT plates, treated with PK (0.5 µg ml^−1^ for 90 min at 37°C), denatured, and infected (PrP^Sc+^) cells were detected by immunocytochemistry using alkaline phosphatase-conjugated POM1 mouse anti-PrP and an alkaline phosphatase-conjugated substrate kit (Bio-Rad). We performed serial tenfold dilutions of experimental samples in cell culture medium containing healthy mouse brain homogenate. Scrapie-susceptible PK1 cells were then exposed to dilutions of experimental samples ranging from 10^−4^ to 10^−6^ (corresponding to homogenate with a protein concentration of 10 µg ml^−1^ to 0.1 µg ml^−1^), or to a 10-fold dilution of RML or healthy mouse brain homogenate. Samples were quantified in endpoint format by counting positive wells according to established methods [23].

### Statistical analysis

One-way ANOVA with Tukey's post-test for multi-column comparison or a Dunnet's post-test for comparison of all columns to a control column were used for statistical analysis of experiments involving the comparison of three or more samples. Paired Student's t-test was used for comparing two samples. Results are displayed as the average of replicas ± s.d.

### Accession numbers

UniprotKB Reference Sequence: Beta-actin - P60710, Caspase-3 - P70677, CD68 - P31996, Fodrin (αII-spectrin) - P16546, GABA_Aα6_ - P16305, GFAP - P03995, MBP - P04370, MCP-1 - P10148, NeuN - Q8BIF2, PrP27-30 - P04925, Rantes - P30882, TNF - P06804.

## Results

**Figure 1:**
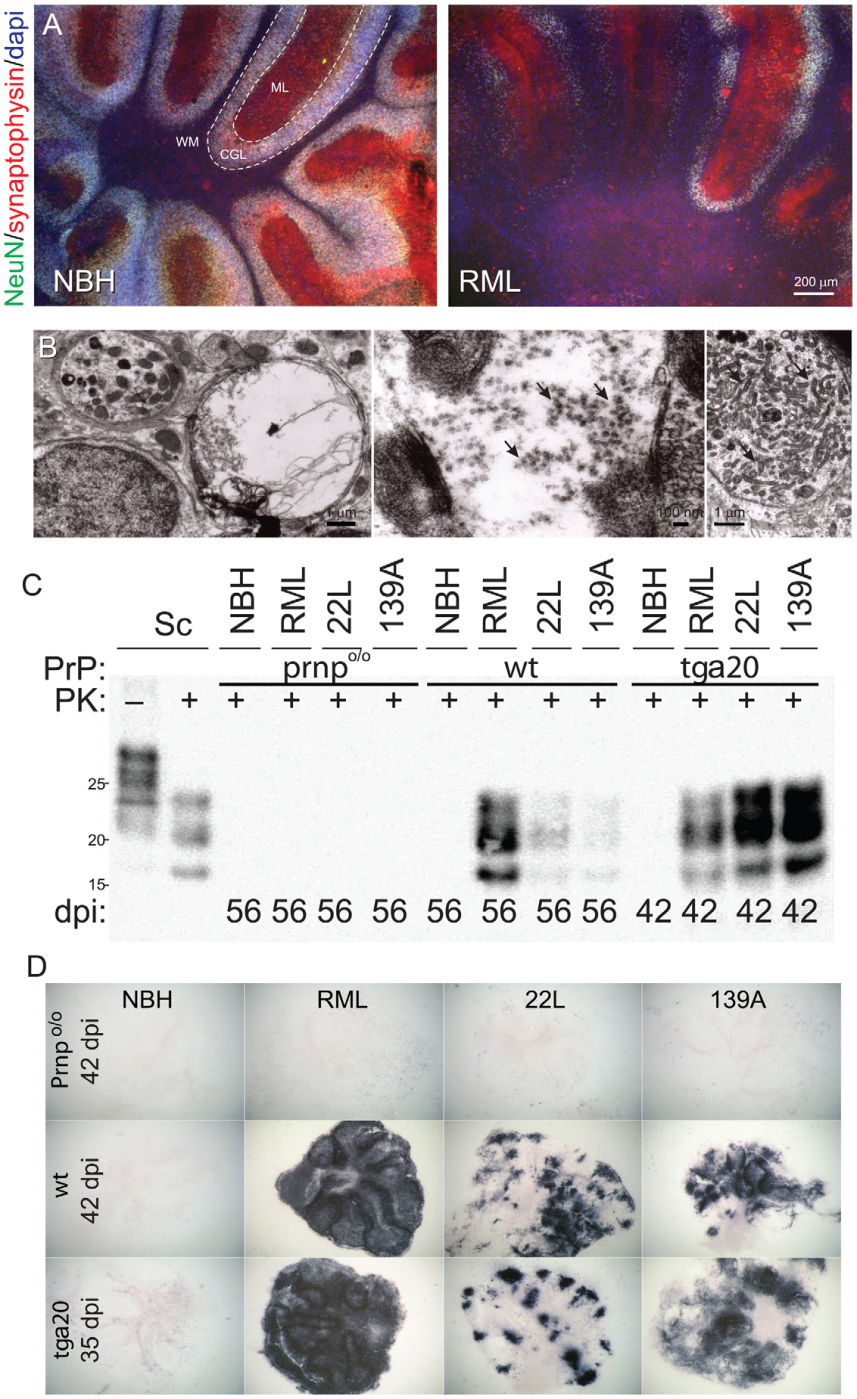
Localization and consequences of prion replication in COCS. (**A**) Fluorescence micrographs showing profound loss of NeuN^+^ cerebellar granule cells and of synaptophysin in wt COCS challenged with RML prions (56 dpi, right panel). No neuronal damage was detected in COCS exposed to non-infectious brain homogenate (NBH; left panel). WM: white matter. ML: molecular layer. CGL: cerebellar granule cell layer. (**B**) Electron microscopy showing membrane-enclosed intraneuronal spongiform vacuoles (left), tubulovesicular structures (PrP-deficient spheres measuring between 20 and 40 nm in diameter, arrow, middle), and degenerating axons accumulating intra-cellular organelles including mitochondria (arrow, right) in RML-infected wt slices at 56 dpi. (**C**) Immunoblots showing PrP^Sc^ in tga20 and wt, but not Prnp^o/o^ COCS exposed to prions (RML, 22L, 139A) or NBH. Sc; scrapie-sick wt mouse brain homogenate, used as positive control. (**D**) Histoblots showing strain-specific differences in PrP^Sc^ deposition patterns of tga20 and wt COCS. No PrP^Sc^ signal was observed in Prnp^o/o^ COCS and in PrP-expressing COCS exposed to NBH.

In a first series of experiments, we confirmed our published finding that Rocky Mountain Laboratory (RML) prions replicate efficiently in COCS and, in addition, we found that infectivity could be serially passaged by slice-to-slice transmission (Figure S1). We then exposed wild-type (*wt*) COCS to RML, and maintained them for 56 days post-inoculation (dpi). COCS exposed to equivalent amounts of non-infectious brain homogenate (NBH) maintained a stable density of NeuN^+^ cerebellar granule cells (CGC) throughout the observational period (Figure 1A). In contrast, the cerebellar granule layer (CGL) of prion-infected cultures displayed progressive loss of synaptophysin and NeuN (Figure 1A). Similarly, RML-treated PrP^C^-overexpressing *tg*a*20* [16] cultures showed a loss of CGL and calbindin^+^ Purkinje cells (PC) at 42 dpi, whereas NBH treated slices maintained immunohistochemically intact CGL and PC (Figure S2A, D). Astrogliosis seemed to be enhanced in prion-infected COCS as observed by EM (Figure S2B), although this finding was partially obfuscated by the high level of basal gliosis present in all COCS. TSE-typical spongiform changes with a vacuole size of 2–5 µm, tubulovesicular structures and dystrophic neurites all were exclusively present in prion-infected *wt* (Figure 1B, Table 1) and *tg*a*20* cultures (Figure S2C, S3A).

**Table 1:** EM quantification of vacuoles and degenerating neurites.

	NBH	RML	22L	139A
**Grid squares counted**	325	211	121	177
**Vacuoles**	0	43 (15*)	36 (20*)	58 (15*)
**Degenerating neurites**	0	48	15	41
**Tubulovesicular structures**	not present	present	present	present

* Number of myelinated vacuoles.

Grid size was 1225 µm^2^ and each grid square (61.25 µm^2^) contained a maximum of one vacuole or degenerating neurite. A fraction of the vacuoles were myelinated as indicated.

**Figure 2:**
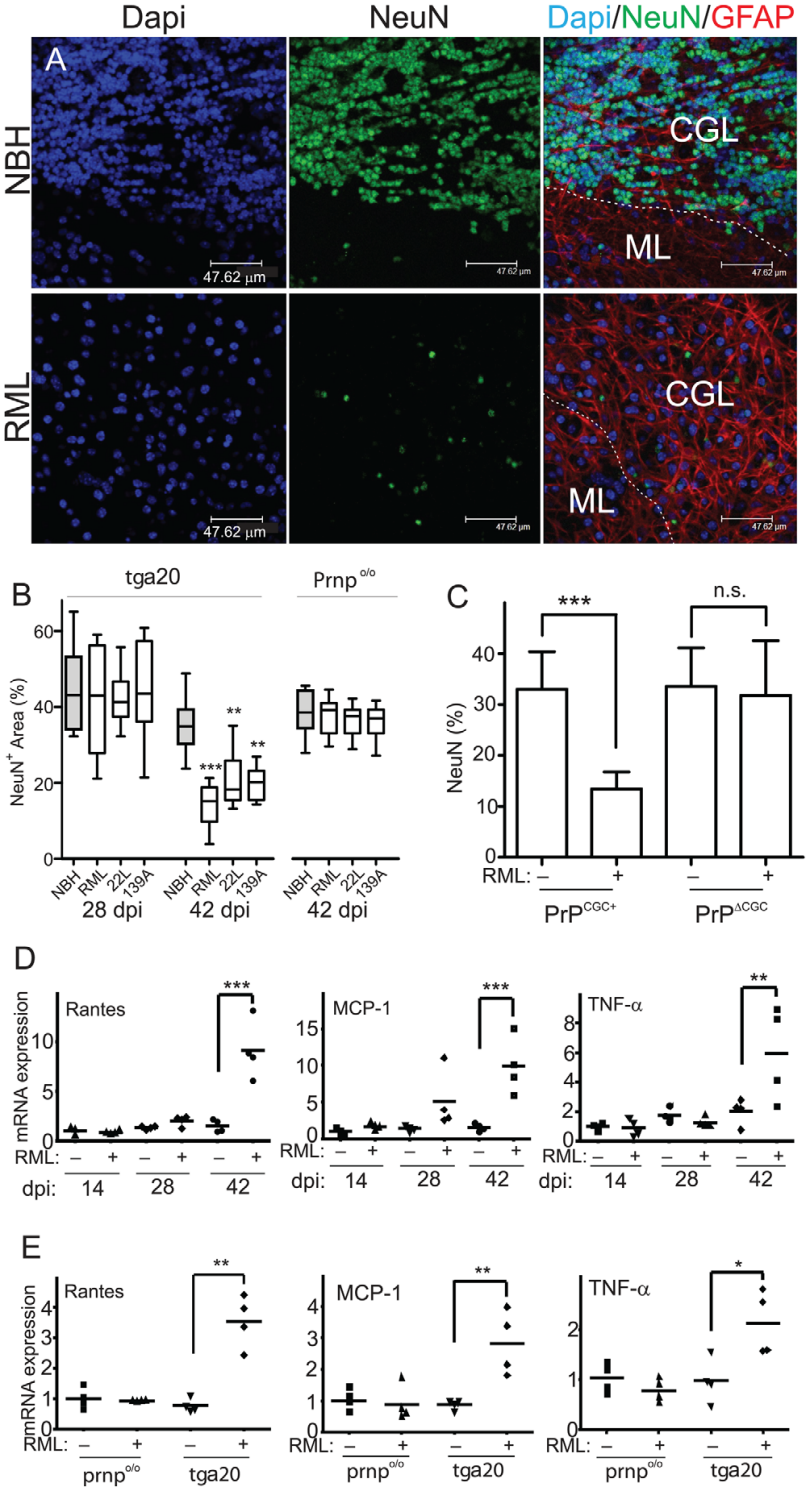
Prion-induced neurodegeneration ex vivo. (**A**) COCS were stained with IgG1 antibodies to NeuN (green) and GFAP (red) and counterstained with DAPI (blue). Representative images were recorded by confocal laser-scanning microscopy approximately 5 µm below the tissue surface using a 40× oil-immersion lens. Prion infection elicited severe NeuN^+^ cell loss. (**B**) Morphometric quantification of NeuN coverage in COCS prepared from tga20 and Prnp^o/o^ mice. The loss of NeuN immunoreactivity was progressive over time (left graph). No NeuN loss was seen in PrP^C^-deficient COCS (right graph). Statistical shorthand here and henceforth: *: p<0.05; **: p<0.01; ***: p<0.001. (**C**) In contrast to control COCS (PrP^CGC+^), COCS prepared from mice with conditional PrP-ablation in CGCs (PrP^ΔCGC^) showed no prion toxicity at 56 dpi. (**D**) Tga20 cultures exposed to NBH (−) or RML (+) were analyzed at various time points by quantitative reverse-transcription PCR (qPCR) for Rantes, MCP-1 and TNFα. ΔCt values were normalized to actin, with 1 being the ratio in uninfected cultures at 14 dpi ± s.d. Each data point is the average of 4 biological replicas. (**E**) Tga20 and Prnp^o/o^ cultures treated with NBH (−) or RML (+) were harvested at 42 dpi and analyzed by qPCR for Rantes, MCP-1 and TNFα. ΔCt values were normalized to actin, with 1 being the ratio in untreated Prnp^o/o^ COCS.

*Tg*a*20* COCS did not exhibit overtly altered GFAP^+^, MBP^+^, and CD68^+^ cells (Figure 2A, S2A, D). Residual NeuN^+^ cells were frequently associated with microglia, suggesting phagocytosis of dying cells (Figure S2E). Hence the defining hallmarks of scrapie, including selective neuronal loss, vacuolation and astrogliosis were reproduced in prion-infected COCS.

### Strain-specific effects of prion infection

We exposed COCS prepared from *tg*a*20* and *Prnp* ^o/o^ mice to the three distinct prion strains, RML, 22L, and 139A. At 42 dpi, PrP^Sc^ was detected in *tg*a*20* COCS exposed to each strain (Figure 1C), but neither in *Prnp* ^o/o^ COCS nor in NBH-exposed *tg*a*20* COCS, confirming that COCS are infectible with many different prion strains, and that PrP^Sc^ reflected de novo synthesis rather than residual inoculum. Similar results were obtained for *wt* COCS at 56 dpi (Figure 1C), although the lower PrP^C^ expression resulted in lower PrP^Sc^ levels (Figure S2F).

Different prion strains induce distinct patterns of PrP^Sc^ deposition and lesion profiles, and can differentially target distinct neuronal subpopulations. Histoblots of COCS revealed strikingly strain-specific PrP^Sc^ deposition patterns. RML induced a diffuse signal akin to the “synaptic” pattern seen *in vivo*; 22L induced a plaque-like pattern with dense, multifocal deposits; and 139A induced ubiquitous PrP^Sc^ patches except in the central white matter (Figure 1D). Prion-infected *wt* COCS at 42 dpi displayed patterns equivalent to those found in *tg*a*20* COCS at 35 dpi. No signal was seen in histoblots of prion-challenged *Prnp* ^o/o^ and NBH-challenged *tg*a*20* COCS (Figure 1D).

RML infected *tg*a*20* COCS showed a selective loss of NeuN^+^ cells at 42 dpi (Figure 2A). NeuN^+^ cell loss was undetectable at 28 dpi, and was absent from *Prnp* ^o/o^ COCS at 42 dpi, but was conspicuous and significant in RML-infected COCS at 42 dpi (Figure 2B). Therefore neurodegeneration was driven by prion replication rather than by toxic inoculum constituents. The severity of the spongiform changes was similar to that of cell loss: RML, 22L, and 139A-infected *wt* COCS (56 dpi) displayed vacuoles in 20, 30, and 33% of all EM grid squares respectively, whereas no vacuolation occurred after NBH challenge (Table 1). Many of these vacuoles were myelinated, consistent with intraaxonal localization. RML, 22L, and 139A infected *wt* COCS (56 dpi) displayed dystrophic neurites in 23, 12 and 21% of all grid squares respectively, with no degenerating neurites observed in NBH samples (Table 1). Tubulovesicular structures were sporadically observed in all strains, but never in NBH samples (Table 1).

Selective ablation of PrP on cerebellar granule neurons (CGCs) using GABA_Aα6_-cre mice [14] intercrossed to PrP tg37 mice [15] (PrP^ΔCGC^) resulted in abrogation of RML-induced loss of NeuN^+^ cells, showing that neuronal PrP confers neurotoxic signaling (Figure 2C, S3B). Concomitantly with cell loss, a strong induction of inflammatory cytokines TNFα, MCP-1, and Rantes was observed at 6 weeks post-inoculation in RML *tg*a*20* cultures, but not in RML-treated *Prnp* ^o/o^ COCS [5] (Figure 2D–E). Samples in Figure 2D were normalized to *tg*a*20* NBH samples at 14 dpi and samples in Figure 2E were normalized to NBH treated *Prnp* ^o/o^ samples using the ΔΔ_Ct_ method. The inflammatory mediators were found to be upregulated over NBH-exposed slices across 4 independent sets of samples analyzed by real-time PCR or microarray analysis, each with 4 biological replicas per group (data not shown).

### Compounds conferring protection against prions

**Figure 3:**
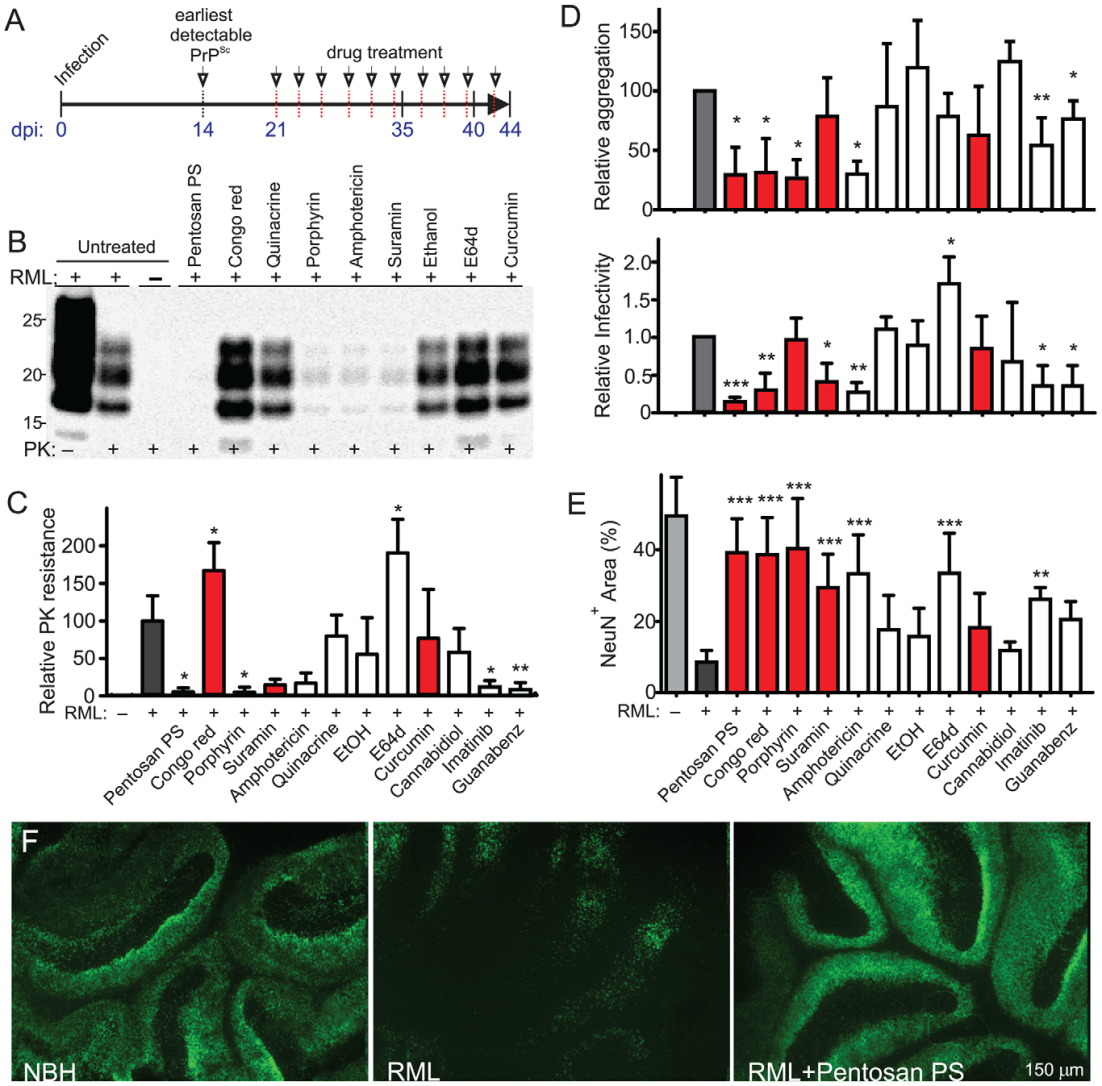
Anti-prion compounds. (**A**) Timeline of the pharmacological experiments in *tg*a*20* COCS. Drug treatments were initiated at 21 dpi and re-added with each medium change (arrows). Longitudinal analyses revealed no cell loss at <35 dpi. Prion replication was assessed at 35 dpi, actively ongoing cell death at 39–42 dpi (PI; fodrin; TUNEL) and severe neuronal loss at 42–45 dpi (NeuN). (**B**) Representative PrP^Sc^ immunoblots of *wt* COCS challenged with RML prions (+) or NBH (−) and treated with compounds at 21–35 dpi. Blots were probed with POM1. (**C**) Total PrP^Sc^ (all PrP^Sc^ bands) was quantified densitometrically and compared to untreated RML-infected COCS (grey). Red: compounds which were reported to interact with PrP^C^ or PrP^Sc^. (**D**) Misfolded protein assay (upper panel) and scrapie cell assay (lower panel) of all *wt* samples from C. Relative aggregation and infectivity: values were compared to untreated RML-infected COCS ± s.d. (**E**–**F**) NeuN morphometry (lower panel) of *tg*a*20* slices exposed to RML or NBH and treated with compounds from 21–42 dpi. All compounds reducing aggregation and/or infectivity were neuroprotective except guanabenz. In contrast, E64d was neuroprotective although it enhanced prion infectivity. (**F**) NeuN staining showing CGL damage by RML infection (middle) and its prevention by pentosan polysulphate (right).

Several compounds reported to abrogate prions from infected cell lines were tested on *wt* COCS for their ability to suppress prion deposition. In order to study their potential to ameliorate prion neurotoxicity, instead, we opted to use *tg*a*20* COCS because they showed accelerated cell loss and smaller interslice variability. In order to distinguish between interference with prion replication and prevention of initial infection, drug treatment was initiated at 21 dpi (Figure 3A) when PrP^Sc^ accumulation was already conspicuous [13]. At 35 dpi, before the appearance of neurotoxicity, PrP^C^ and PrP^Sc^ were measured in *wt* samples by Western blotting (Figure 3B–C, S4A, *n* = 2–3). In addition, we measured protein aggregation by the misfolded protein assay (MPA), which selectively captures PrP aggregates and upon a limited trypsin digestion returns quantitative responses over a 4-log range [19] (Figure 3D, S4B; *n* = 4). Prion titers were determined by the scrapie cell assay in end-point format (SCEPA; Figure 3D; *n* = 3) [23]. Finally, drug-treated *tg*a*20* COCS were maintained until 42 dpi and analyzed by NeuN morphometry (Figure 3E–F; *n* = 10). Neurotoxicity was defined as significant NeuN^+^ CGC loss over NBH treatment, and neuroprotection was defined as significant NeuN^+^ CGC rescue (*p*<0.05) over infected, untreated COCS. By these criteria, pentosan polysulphate (PPS), amphotericin B, Congo red, porphyrin, suramin, imatinib, and E64d were neuroprotective, with several compounds completely preventing cell loss (Figure 3E). No compounds were toxic to non-infected cultures at the concentration used (Figure S4C).

We then studied the effects of each compound onto PrP^Sc^ accumulation, PrP aggregation, and prion infectivity by quantitative Western blotting after PK digestion, MPA, and SCEPA, respectively. PPS, suramin, amphotericin B, guanabenz and imatinib showed a strongest suppression of infectivity, PrP aggregation, and PK resistance, whereas curcumin, cannabidiol and quinacrine were ineffective (Figure 3C–D). In RML infected drug treated COCS total PrP (PrP^C^+PrP^Sc^) was only marginally affected (Figure S4A). In uninfected cultures the amount of total PrP was unaffected by drug treatment (Figure S4D; *n* = 3). By western blotting, suramin samples showed decreased FL-PrP and E64d samples showed increased FL-PrP, suggesting that E64d affects lysosomal degradation of PrP (Figure S4D). A significant reduction in FL-PrP was observed only for suramin and quinacrine treated cultures by FRET assay (Figure S4E; *n* = 4), suggesting that the anti-prion drug effects affected predominantly PrP^Sc^ (Figure S4A). Surprisingly, Congo red increased PK resistance while suppressing infectivity and aggregation (Figure 3C–D). The dissociation of aggregation and PK resistance from infectivity suggests that Congo red acts differentially on the stability and frangibility of PrP aggregates, as previously described for other amyloidotropic compounds in prion-infected COCS [3], [24]. In agreement with this notion, incubation of RML brain homogenate with Congo red sufficed to confer increased PK-resistance (Figure S4F), while relative aggregation was not significantly affected (Figure S4G). Quinacrine, but no other compound, also afforded partial neuroprotection against 3 mM H_2_O_2_ (Figure S4H).

### Calpains, but not caspases, mediate prion neurotoxicity

**Figure 4:**
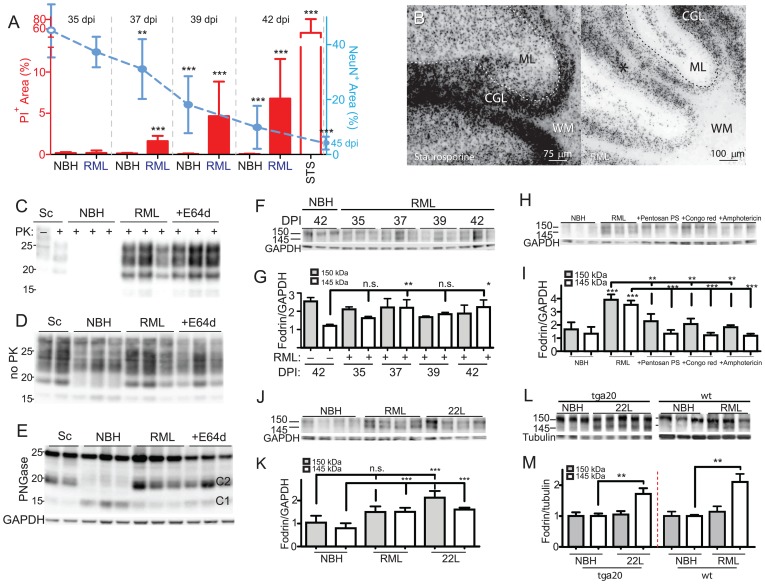
Temporal aspects of prion toxicity. (**A**–**B**) *Tg*a*20* slices were exposed to RML or NBH, cultured and scored for PI incorporation (red bars) and NeuN^+^ morphometry (cyan dashed line) at 35, 37, 39, 42 and 45 dpi (RML; closed circles, NBH 45 dpi; open circle). The progressive appearance of PI^+^ cells coincided with NeuN degradation, suggesting that dying cells were efficiently removed. Sts: staurosporine treatment (48 hrs, 5 µM). (**B**) Fluorescent images of PI-stained COCS (42 dpi). For better contrast, a non-linearly modified negative of the image is shown. PI^+^ cells are mostly located in the CGL. (**C**–**E**) Treatment of *tg*a*20* RML cultures with tool compounds was initiated at 21 dpi. COCS were harvested at 39 dpi (*n* = 3). Homogenates were treated with PK (**C**), left untreated (**D**) or treated with PNGase (**E**), and Western blots were probed with POM1 to detect PrP^Sc^, total PrP, or total unglycosylated PrP (full-length and C1/C2 proteolytic fragments). The GAPDH band in panel E is representative for D, and samples were loaded as in panel C. (**F**) Western blots of infected *tg*a*20* COCS (35–42 dpi; *n* = 3) showing increased 145 kDa α-fodrin cleavage. (**G**) Densitometric quantitation of α-fodrin cleavage fragments shown in panel F (ordinate: ratio of α-fodrin/GAPDH). α-Fodrin cleavage peaked at 37–42 dpi. (**H**–**I**) Western blots (**H**) and densitometric analysis (**I**) of infected *tg*a*20* COCS treated with anti-prion compounds (21–40 dpi) showing increasing 150/145 kDa α-fodrin cleavage in RML samples and reversal by anti-prion compounds. Densitometry readings were normalized against GAPDH. (**J**–**K**) Western blots (**J**) and densitometric analysis (**K**) of NBH, RML and 22L treated *tg*a*20* COCS at 42 dpi, showing significantly increased 145 kDa α-fodrin cleavage in RML and 22L samples. Densitometry readings were normalized against GAPDH. (**L**–**M**) Western blots (**L**) and densitometric analysis (**M**) of brain tissue from terminally sick 22L-infected *tg*a*20* mice and NBH-injected mice (left half of graph) or RML and NBH-treated *wt* mice (right half), showing significantly increased 145 kDa α-fodrin cleavage in infected samples. The left panel of consist of two cropped strips, cropped from the same blot at the same exposure. Densitometry readings were normalized against tubulin and normalized to NBH samples.

Prion-infected COCS displayed TUNEL^+^ (136±53 vs. 19±13 cells/mm^2^; *p* = 0.0014; *n* = 10, Figure S5A–B) and propidium-iodide retaining (PI^+^) cells (Figure 4A–B; *n* = 10), although to a much smaller extent than staurosporine treated COCS (48 h; >2000 TUNEL^+^ cells/field, Figure S5A–B). The progressive increase in PI^+^ cells between 35–42 dpi correlated temporally with NeuN^+^ cell loss (Figure 4A; *n* = 10). Although there was some variability between individual brain slices, all infected cultures showed severe damage at later incubation times (Figure S6A–B). PI^+^ cells were mostly confined to the CGL (asterisk), whereas staurosporine induced rapid and widespread cell death also affecting the CGL (Figure 4B). Hence prion-induced cell death was mostly apoptotic, chronic-progressive rather than acute, and preferentially targeting the CGL.

Whilst PPS, Congo red, and amphotericin B counteracted neurotoxicity by inhibiting prion replication, E64d prevented neurotoxicity, yet it did not reduce MPA readings, and enhanced prion titers and PrP^Sc^ deposition (Figure 3E), suggesting that it interfered with neurotoxic events downstream of prion replication. Accordingly, E64d did not affect PrP^Sc^ glycosylation (Figure 4C), total PrP expression and running pattern (Figure 4D), and PrP processing into C1 and C2 fragments in prion-infected COCS, although a reduction in full-length PrP was observed (Figure 4E, S7).

Because E64d inhibits cystein proteases including calpains, we investigated a possible involvement of calpains in neurotoxicity. Both calpains and caspases can cleave α-fodrin into a 150 kDa fragment. In addition, calpains selectively generate a diagnostic 145 kDa fragment whereas caspases give rise to a 120 kDa fragment [25]. Faint 145 kDa α-fodrin bands were barely apparent in uninfected COCS, but displayed increased intensity upon prion infection (*n* = 3; *p*<0.01 at 37 dpi), peaking at 37–42 dpi on a timescale consistent with increased PI incorporation (Figure 4A, F, G). Therefore, enhanced α-fodrin cleavage generating the 145 kDa fragment accompanies prion-induced neurodegeneration, suggesting calpain activation. Fodrin cleavage was counteracted by inhibiting prion replication with anti-prion compounds PPS, congo red, and amphotericin B (Figure 4H–I; *n* = 3; *p*<0.001) and was also induced by a second prion strain, 22L (Figure 4J–K; *n* = 4, *p*<0.05–0.01). The 145 kDa fodrin cleavage product was also increased in brains of terminally sick 22L infected *tg*a*20* mice and RML infected *wt* mice, suggesting prion-induced calpain activation *in vivo* (Figure 4L–M; *n* = 3–5, *p*<0.01).

**Figure 5:**
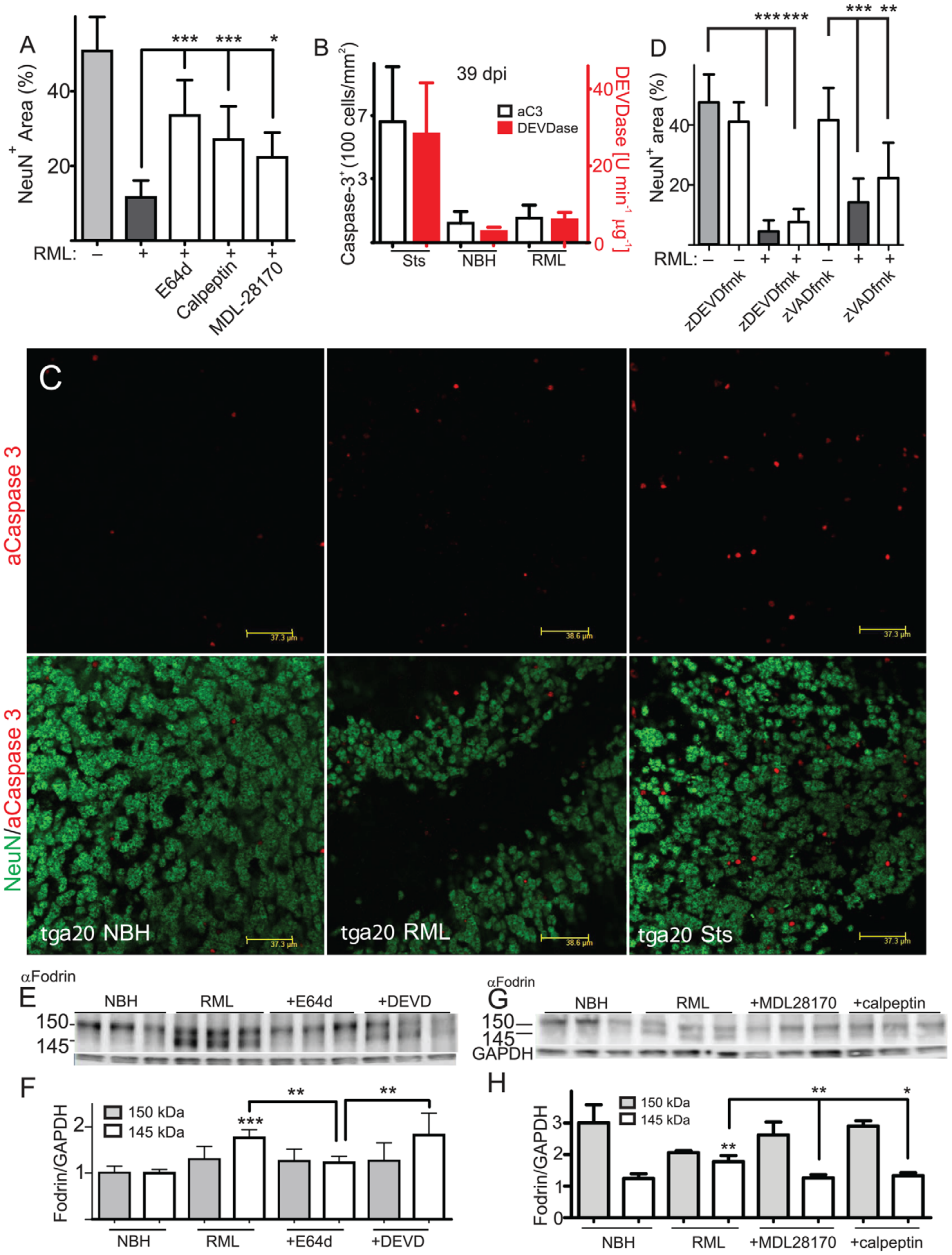
Calpain-mediated prion toxicity. (**A**) Treatment of RML infected *tg*a*20* COCS with three calpain inhibitors from 21–44 dpi significantly antagonized neuronal loss in prion-infected *tg*a*20* COCS. Grey bars: untreated NBH (−) and RML (+) samples. (**B, C**) *Tg*a*20* cultures were harvested at 39 dpi and stained for activated caspase-3 (white bars) or homogenized and assayed for DEVDase activity (red bars). In contrast to staurosporine treatment (STS; 24 h), prion infection did not significantly increase DEVDase and aC3+ cells. Average DEVDase activity in COCS (4 pools of 20 slices each) normalized to the protein amount ± s.d. (**C**) Representative images of B. (**D**) *Tg*a*20* slices were treated with z-DEVD-fmk or zVAD-fmk from 21–44 dpi and analyzed by morphometric analysis. Grey bars: untreated. (**E**–**F**) *Tg*a*20* cultures were treated with calpain and caspase inhibitors from 21–41 dpi, and probed for α-fodrin (*n* = 6–7). GAPDH: loading control. (**F**) Densitometric quantification showing that α-fodrin cleavage was enhanced by RML prion infection, and suppressed by E64d, but not by caspase inhibition (DEVD). (**G**–**H**) *Tg*a*20* cultures were treated with calpain inhibitors as in E, and probed for α-fodrin (*n* = 3). GAPDH: loading control. (**H**) Densitometric quantification showing that α-fodrin cleavage was enhanced by RML prion infection, and suppressed by MDL-28170 and calpeptin.

We then sought to dissect the relative contribution of calpain and caspases to COCS neurotoxicity. Two further calpain inhibitors, MDL-28170 and calpeptin, also significantly reduced prion neurotoxicity (Figure 5A, S8A; *n* = 9). Conversely, the prevalence of cells containing activated caspase 3 (aC3) was similar in uninfected vs. prion-infected COCS (79±53 vs 139±53 cells/mm^2^; *n* = 8; *p*>0.05), and enhanced by staurosporine treatment (24 h; 660±295 cells/mm^2^; *p*<0.01; Figure 5B–C). Also, DEVDase activity in COCS homogenates did not increase significantly upon prion infection (Figure 5B; *n* = 4, *p*>0.05). Crucially, the caspase inhibitors z-DEVD-fmk and z-VAD-fmk antagonized staurosporine-induced toxicity (Figure S8B), yet neither compound conferred antiprion neuroprotection (Figure 5D; *n* = 10).

We then treated prion-infected COCS with protease inhibitors starting at 21 dpi, and harvested samples at 41 dpi. The prion-dependent increase in α-fodrin cleavage was reduced by E64d (*n* = 7, *p*<0.01), calpeptin and MDL-28170 treatment (*n* = 3, *p*<0.05–0.01), but not by caspase inhibition by z-DEVD-fmk (Figure 5E–H; *n* = 6, *p*>0.05). The above results imply that calpains, rather than caspases, are causally involved in prion-induced α-fodrin cleavage and neurotoxicity.

## Discussion

Prion infection of COCS faithfully reproduced all salient features of the pathogenesis of prion diseases: (1) progressive, profound neuronal loss after a protracted asymptomatic incubation time (42 dpi in *tga20* COCS), (2) a proinflammatory glial response with vigorous upregulation of Rantes, MCP-1 and TNFα, (3) typical neuropathological changes such as spongiform changes, tubulovesicular structures, and neuroaxonal dystrophy, and (4) an excessive meshwork of astrocytic processes exceeding that observed in controls and reminiscent of gliosis. Conversely, CGC loss in COCS was faster and stronger than in prion-infected animals [26], suggesting that prion clearance may be less efficient in COCS than *in vivo*.

Compounds suppressing prion replication and/or interacting with PrP^Sc^ (PPS, CR, porphyrin, amphotericin B, imatinib and suramin) were neuroprotective in the COCS-based screen, whereas compounds previously reported to be effective in prion-infected cell lines but not *in vivo* (quinacrine [27], [28], curcumin [29], and cannabidiol [30]) were ineffective despite the report of a direct interaction of curcumin with PrP^C^ (Table S1 in Text S1). Hence inhibition of prion replication was neuroprotective to COCS, and the COCS neurodegeneration assay predicted *in vivo* efficacy more accurately than cell-based assays.

Not all drugs acted in a perfectly consistent manner in all assays – a fact which reflects the biophysical and biological differences between the variables measured by each assay. In particular, Guanabenz treatment decreased prion infectivity, but failed to show neuroprotection. This may reflect subliminal drug toxicity, which indeed became evident after treatment of COCS with higher concentrations of guanabenz (data not shown).

Congo red increased the protease resistance of PrP, yet it decreased the capture of protein aggregates and prion infectivity. This is consistent with results from cultures treated with other amyloid-binding compounds [3], [24] and probably indicates that these compounds hinder prion replication by hyperstabilizing protein aggregates. Indeed, congo red was reported to regulate the stability of PrP^Sc^ aggregates [31] and we found that PK resistance of PrP^Sc^ was enhanced by Congo red treatment. Suramin yielded slightly higher MPA readings than what might have been expected from PrP^Sc^ and infectivity determination, suggesting that it might partially unfold prion fibrils and make them more available for MPA capture. Porphyrin reduced PrP^Sc^ and MPA readings, and was neuroprotective, yet it did not affect prion titers. These discordant findings suggest that porphyrin may render PrP^Sc^ less toxic without appreciably reducing prion replication.

E64d was neuroprotective to COCS despite slightly enhanced PrP^Sc^ and infectivity accumulation, suggesting blockade of neurotoxic pathways downstream of prion replication. E64d inhibits preferentially cathepsins B, H, and L as well as calpains, which participate to cell death [32] in excitotoxicity [33], brain ischemia [34] and Alzheimer's disease [35]. Further calpain inhibitors (calpeptin and MDL-28170) were also neuroprotective *in vitro* and all blocked the calpain-specific cleavage fragments of the substrate fodrin. Instead of reducing prion replication or C2 cleavage [36], E64d enhanced prion accumulation in COCS, possibly by inhibiting their lysosomal degradation.

Caspases can be cleaved by calpains [37], and prion-infected brains can contain scattered caspase-3^+^ and TUNEL^+^ cells [38]. Although prion-infected COCS also harbored TUNEL^+^ cells, we failed to detect any caspase activity, any activated caspase-3, and any caspase-dependent α-fodrin cleavage. Crucially, two distinct caspase inhibitors failed to confer neuroprotection. These data suggest that prion neurotoxicity is calpain-dependent but caspase-independent in CGCs. The prevalence of PI^+^ cells rose rapidly at the time of onset of enhanced α-fodrin cleavage and was closely followed by neuronal loss, suggesting that calpain-driven cell death was quickly followed by lysis and removal.

Calpain activation strongly suggests that exaggerated calcium influx may represent an important upstream event in pathogenesis. We have attempted to test the latter hypothesis directly, but the slow progression of prion pathology in COCS (which is similar to that observed *in vivo*) posed significant obstacles. In particular, we found that chronic treatment of COCS with Ca^2+^ chelators or protracted culture in low-calcium medium was too toxic to allow for any firm conclusion (data not shown).

We here identify calpain inhibition as an experimental paradigm that uncouples prion replication from neurotoxicity. While a few other examples of retarded neuropathogenesis despite florid prion replication where reported earlier by us and others [39], [40], the molecular mechanism of such uncoupling had remained unclear. The data presented here suggest that uncoupling occurs because calpain is a crucial link between prion-induced intracellular hypercalcemia and cell death. It will be exciting to test whether the chain of event hypothesized above may be manipulated in order to control prion-induced damage rather than prion replication.

Beyond the biological phenomena described above, a significant advance provided by this study consists - in our view - of providing a convenient experimental paradigm that combines the exquisite accessibility of *in vitro* systems with the rich palette of neurotoxic and neurodegenerative events characteristic of prion diseases, such as spongiform changes, neuronal loss, and astrogliosis. The latter features could hitherto only be studied in prion-infected experimental animals, since prion-infected cell lines do not exhibit significant cytotoxicity. The successful transposition of prion-specific neurodegenerative features to cultured tissues does away with many issues of pharmacokinetics, bioavailability, and animal welfare, thereby enabling a broad range of pharmacological experiments that had been hitherto impractical or impossible. It is to be hoped that many laboratories will make use of the technologies described here, and that neurodegenerative prion science will consequently progress at a faster pace.

## Supporting Information

Figure S1 *Tg*a*20* COCS were infected with RML and harvested at 35 dpi (passage 1). COCS homogenate was re-transmitted into *tg*a*20* COCS at 10 µg ml^−1^, equivalent to a titer of 10^−4^, and harvest at 35 dpi (passage 2). Immunoblots of PrP^Sc^ in *tg*a20 COCS show an efficient prion replication at both the first and the second passage through COCS. Sc; scrapie-sick *wt* mouse brain homogenate.
(EPS)

Figure S2 (**A**) *Tg*a*20* RML COCS were stained for calbindin (green) and MBP (red) at 42 dpi. Images were recorded by fluorescence (4× lens). (**B**) EM image of reactive astrogliosis in RML-infected *tg*a*20* slices at 39 dpi. Reactive astrocytes are characterized by prominent intermediate filament, giving the appearance of intracellular lamellar structures (arrow). (**C**) Electron microscopy showing membrane-enclosed intraneuronal spongiform vacuoles (left), tubulovesicular structures (arrows, middle) and degenerating axons accumulating intra-cellular organelles including mitochondria (arrow, right) in RML-infected *tg*a*20* slices at 39 dpi. (**D–E**) *Tg*a*20* slices were prepared as in (A), and stained for NeuN (green), anti-CD68 (red), and optionally DAPI (blue). Images were recorded by fluorescence (4× lens, **D**) or confocal microscopy (40× oil lens, **F**, 5 µm below the tissue surface). (**F**) Immunoblot of total PrP (PrP^C^ + PrP^Sc^) from *tg*a*20*, *wt* and *Prnp* ^o/o^ slices exposed to prion-containing brain homogenates (RML, 22L or 139A) or NBH, cultured for 42 (*tg*a*20*) to 56 days (*wt* and *Prnp* ^o/o^). Membranes were probed with anti-PrP antibody POM1.
(EPS)

Figure S3 (**A**) Histology of NBH and RML treated *tg*a*20* COCS at 42 dpi. H&E stainings identified vacuolation selectively in prion infected tissue (arrow). (**B**) Representative images of Figure 2C. COCS prepared from mice with conditional PrP-ablation in CGCs (PrP^ΔCGC^) showed no prion toxicity at 56 dpi, while control COCS (PrP^CGC+^) showed prion toxicity.
(EPS)

Figure S4 (**A**) Immunoblot of C57BL/6 COCS exposed to RML-infected (RML) or uninfected (NBH) brain homogenate, cultured for 21 days, treated with various compounds for 14 days, and probed with antibody POM1 to PrP at 35 dpi. Leftmost lane: brain homogenate of a terminally scrapie-sick *wt* mouse. (**B**) Misfolded protein assay (MPA) of RML homogenate decadically diluted in NBH, with or without trypsin digestion. Trypsin digestion abolished NBH background signal, while retaining the specific RML signal. (**C**) Morphometric assessment of NeuN^+^ area in *tg*a*20* slices exposed to NBH (−) and treated with various compounds for 21 days starting at 21 dpi. (**D**) Immunoblot and morphometric analysis of C57BL/6 COCS exposed to uninfected (NBH) brain homogenate, cultured for 21 days, treated with various compounds for 14 days, and probed with antibody POM1 to PrP at 35 dpi. Results are presented as the ratio between PrP^C^ and actin, and data points were normalized to untreated samples. (**E**) FRET analysis of FL-PrP expression in samples from D. (**F**) RML brain homogenate (1 µg µl^−1^) was incubated with different concentrations of congo red for 1 h at 37°C, PK-digested, probed for PrP and analyzed by morphometry. Treatment with Congo red increased the amount of total PrP and the PK-resistance of RML. (**G**) Samples from F were analyzed by MPA. No significant changes were observed in Congo red-treated samples. (**H**) *Tg*a*20* cultures were kept for 14 days, pre-treated for 30 min with anti-prion compound and then exposed to 3 mM H_2_O_2_ for 48 h. Propidium iodide incorporation was assessed by morphometric analysis and data are presented as the average PI^+^ area of 10 slices ± s.d. Light grey bars: no treatment; dark grey: H_2_O_2_ followed by no pharmacological treatment; red: treatment with anti-prion compounds known to directly interact with PrP.
(EPS)

Figure S5 *Tg*a*20* slices were exposed to RML or NBH, cultured and analyzed at 42 dpi by TUNEL assay and counterstained with dapi. Sts: staurosporine treatment (48 hrs, 5 µM). (**A**) Representative TUNEL images. (**B**) The number of TUNEL+ cells was counted and RML treated samples showed increased TUNEL positivity compared to NBH samples and substantially less than STS samples.
(EPS)

Figure S6 (**A**) *Tg*a*20* slices exposed to RML or NBH at 45 dpi from Figure 4A. NeuN images represent the slice with the highest and lowest NeuN value quantified in Figure 4A. (**B**) Images of strongest and weakest PI-positive image from Figure 4A at 42 dpi.
(EPS)

Figure S7 Densitometric analysis of PrP band on Figure 4E, normalized to GAPDH. RML infected COCS showed increased density of C2 and decreased density of C1 fragments. While E64d had no significant impact on C1 or C2 fragments, a small decrease was observed in full length PrP.
(EPS)

Figure S8 (**A**) *Tg*a*20* slices were treated with calpain inhibitors from 21–42 dpi and analyzed by NeuN morphometry. Uninfected COCS showed no adverse effects of calpain inhibitor treatment. (**B**) Staurosporine treatment of uninfected *tg*a*20* cultures treated with caspase and calpain inhibitors. PI incorporation was assessed after 48 h staurosporine treatment (1 µM) in the presence or absence of drug treatment and analyzed by morphometry (*n* = 9).
(EPS)

Text S1 This file contains Table S1, which shows the effects of anti-prion compounds reported in this study. The ‘*in vitro*’ and ‘*in vivo*’ columns describe the reported abilities of drugs to affect prion replication in prion infected cell lines or mice respectively. ‘PrP interaction’ indicates drugs that are thought to interact physically with PrP^C^ or PrP^Sc^. Drugs were scored as ineffective (0), inhibitory (+), or enhancing (−) for their effect on replication and neuroprotection was scored as ineffective (0) or effective (+).
(DOC)
