# Supplementary material for: Prion Pathogenesis Is Faithfully Reproduced in Cerebellar Organotypic Slice Cultures
Source: PLoS Pathog. 2012 Nov 1;8(11):e1002985. doi: 10.1371/journal.ppat.1002985 (PMC3486912; doi:10.1371/journal.ppat.1002985)
Supplement: Text S1 — This file contains Table S1, which shows the effects of anti-prion compounds reported in this study. The ‘in vitro’ and ‘in vivo’ columns describe the reported abilities of drugs to affect prion replication in prion infected cell lines or mice respectively. ‘PrP interaction’ indicates drugs that are thought to interact physically with PrPC or PrPSc. Drugs were scored as ineffective (0), inhibitory (+), or enhancing (−) for their effect on replication and neuroprotection was scored as ineffective (0) or effective (+). (DOC) [file ppat.1002985.s009.doc]

**Table S1**

|  | *Previously reported* | | | *Reported in this study* | | | | |
| --- | --- | --- | --- | --- | --- | --- | --- | --- |
| Compound | In vitro | In vivo* (i.c.) | PrP interaction |  | PrP^Sc^ | MPA | SCEPA | Neuroprotection |
| Pentosan polysulphate | yes | yes | yes |  | + | + | +++ | +++ |
| Congo red | yes | yes | yes |  | - | + | +++ | +++ |
| Amphotericin B | yes | yes | no |  | 0 | + | +++ | +++ |
| Suramin | yes | n.d. | yes |  | 0 | 0 | + | ++ |
| Imatinib | yes | no | no |  | + | ++ | + | ++ |
| Guanabenz | yes | n.d. | n/a |  | ++ | + | + | 0 |
| Quinacrine | yes | no | no |  | 0 | 0 | 0 | 0 |
| Curcumin | yes | no | yes |  | 0 | 0 | 0 | 0 |
| E64d | yes | no | no |  | - | 0 | - | ++ |
| EtOH | n.d. | n.d. | n/a |  | 0 | 0 | 0 | 0 |
| Cannabidiol | yes | n.d. | no |  | 0 | 0 | 0 | 0 |

* All mice were treated after intracerebral inoculation except imatinib which was used after intraperitoneal prion inoculation.

The number of symbols refer to the degree of statistical power (+ or - = *p* < 0.05; ++ = *p* < 0.01; +++ = *p*< 0.001).

**References**

1. Caughey B, Raymond GJ (1993) Sulfated polyanion inhibition of scrapie-associated PrP accumulation in cultured cells. J Virol 67: 643-650.

2. Doh-Ura K, Ishikawa K, Murakami-Kubo I, Sasaki K, Mohri S, et al. (2004) Treatment of Transmissible Spongiform Encephalopathy by Intraventricular Drug Infusion in Animal Models. J Virol 78: 4999-5006.

3. Caughey B, Brown K, Raymond GJ, Katzenstein GE, Thresher W (1994) Binding of the protease-sensitive form of PrP (prion protein) to sulfated glycosaminoglycan and congo red J Virol 68: 2135-2141.

4. Caughey WS, Raymond LD, Horiuchi M, Caughey B (1998) Inhibition of protease-resistant prion protein formation by porphyrins and phthalocyanines. Proc Natl Acad Sci U S A 95: 12117-12122.

5. Kocisko DA, Caughey WS, Race RE, Roper G, Caughey B, et al. (2006) A porphyrin increases survival time of mice after intracerebral prion infection. Antimicrob Agents Chemother 50: 759-761.

6. Caughey B, Race RE (1992) Potent inhibition of scrapie-associated PrP accumulation by congo red. J Neurochem 59: 768-771.

7. Ingrosso L, Ladogana A, Pocchiari M (1995) Congo red prolongs the incubation period in scrapie-infected hamsters. J Virol 69: 506-508.

8. Mange A, Nishida N, Milhavet O, McMahon HE, Casanova D, et al. (2000) Amphotericin B inhibits the generation of the scrapie isoform of the prion protein in infected cultures. J Virol 74: 3135-3140.

9. Pocchiari M, Schmittinger S, Masullo C (1987) Amphotericin B delays the incubation period of scrapie in intracerebrally inoculated hamsters. J Gen Virol 68: 219-223.

10. Doh-Ura K, Iwaki T, Caughey B (2000) Lysosomotropic agents and cysteine protease inhibitors inhibit scrapie-associated prion protein accumulation. J Virol 74: 4894-4897.

11. Gilch S, Winklhofer KF, Groschup MH, Nunziante M, Lucassen R, et al. (2001) Intracellular re-routing of prion protein prevents propagation of PrP(Sc) and delays onset of prion disease. Embo J 20: 3957-3966.

12. Ertmer A, Gilch S, Yun SW, Flechsig E, Klebl B, et al. (2004) The tyrosine kinase inhibitor STI571 induces cellular clearance of PrPSc in prion-infected cells. J Biol Chem 279: 41918-41927.

13. Yun SW, Ertmer A, Flechsig E, Gilch S, Riederer P, et al. (2007) The tyrosine kinase inhibitor imatinib mesylate delays prion neuroinvasion by inhibiting prion propagation in the periphery. J Neurovirol 13: 328-337.

14. Tribouillard-Tanvier D, Béringue V, Desban N, Gug F, Bach S, et al. (2008) Antihypertensive drug guanabenz is active in vivo against both yeast and mammalian prions. PLoS One 3: e1981.

15. Collins SJ, Lewis V, Brazier M, Hill AF, Fletcher A, et al. (2002) Quinacrine does not prolong survival in a murine Creutzfeldt-Jakob disease model. Ann Neurol 52: 503-506.

16. Caughey B, Raymond LD, Raymond GJ, Maxson L, Silveira J, et al. (2003) Inhibition of protease-resistant prion protein accumulation in vitro by curcumin. J Virol 77: 5499-5502.

17. Hafner-Bratkovic I, Gaspersic J, Smid LM, Bresjanac M, Jerala R (2008) Curcumin binds to the alpha-helical intermediate and to the amyloid form of prion protein - a new mechanism for the inhibition of PrP(Sc) accumulation. J Neurochem 104: 1553-1564.

18. Dirikoc S, Priola SA, Marella M, Zsürger N, Chabry J (2007) Nonpsychoactive cannabidiol prevents prion accumulation and protects neurons against prion toxicity. J Neurosci 27: 9537-9544.
